# Supplementary material for: Enhancer accessibility and CTCF occupancy underlie asymmetric TAD architecture and cell type specific genome topology
Source: Nat Commun. 2019 Jul 2;10:2908. doi: 10.1038/s41467-019-10725-9 (PMC6606583; doi:10.1038/s41467-019-10725-9)
Supplement: Supplementary file 1 — Supplementary Information [file 41467_2019_10725_MOESM1_ESM.pdf]

**Supplementary Information for Barrington et al.**

**“Enhancer accessibility and CTCF occupancy underlie asymmetric TAD architecture and cell type specific genome topology”.**

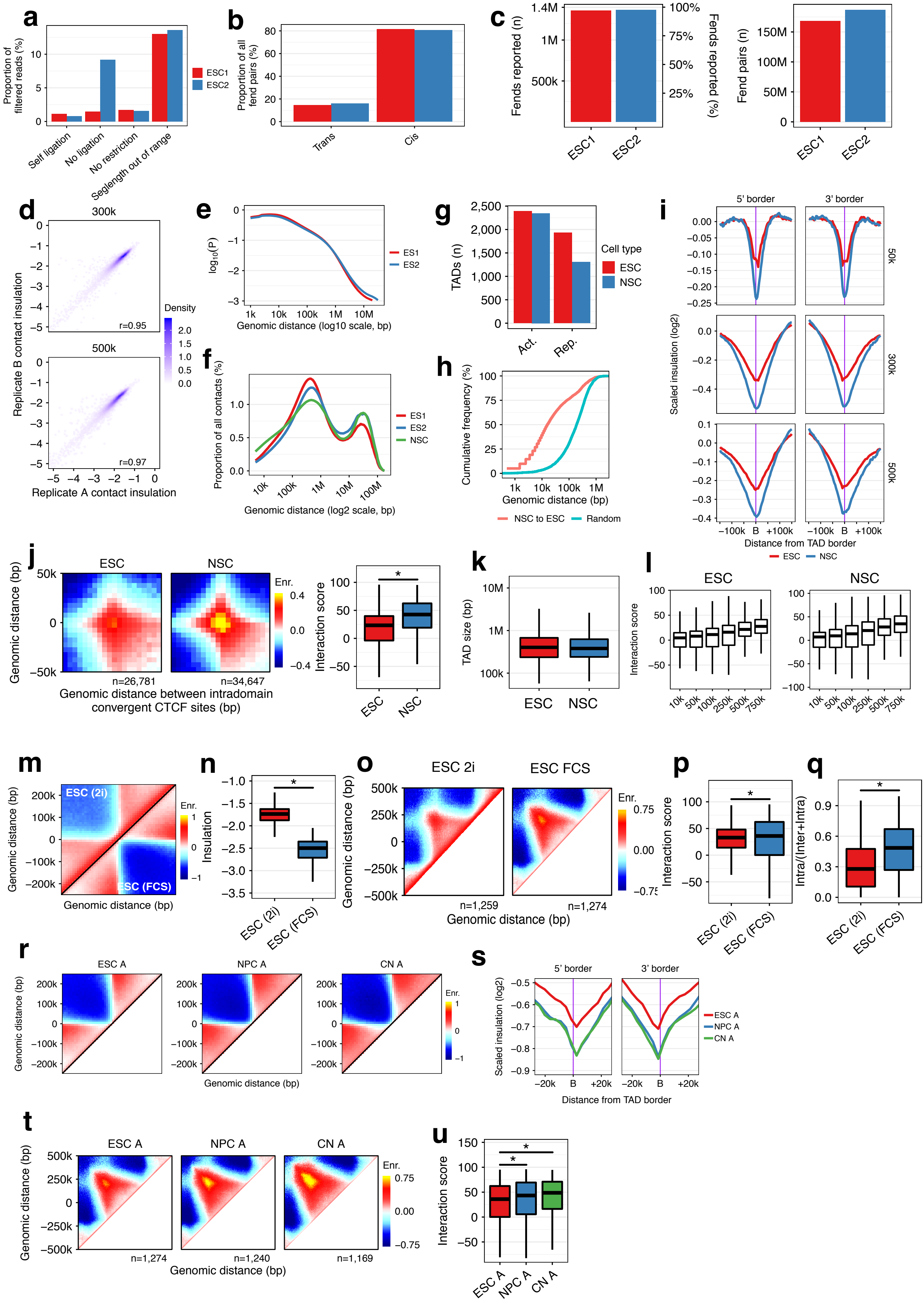


# SUPPLEMENTARY FIGURE 1

**Supplementary Figure 1.** (a) Hi-C dataset quality validation for ESC libraries. Shown are the key statistics on fragment end (f-end) contact pairs that have been extracted from the 'Total Contact Pairs' (Table S1) and used to construct the Hi-C matrices. Reads that reported self-ligation, no ligation, no restriction or mapped too far from a fragment end were removed. (b) An important metric for overall matrix quality is the ratio of cis:trans contacts. The ESC Hi-C matrices prepared for this study represent high quality matrices as indicated by the good ratio between intra- (cis) and inter- (trans) chromosomal interactions for all libraries. (c) f-end coverage is very high and similar in the ESC datasets used. Almost 100% of the 1.4 m valid HindIII fragment ends in mm10 were identified. (d) Pearson's correlation between Hi-C libraries indicates a high reproducibility at several matrix resolutions. Correlation between ESC Hi-C biological replicates, shown at two resolutions (300kb and 500kb) in 20kb bins. Axes units are contact insulation calculated as described in the Methods. (e) and (f) Contact probability distributions. (e) Log-log contact probability as a function of the genomic distance for biological replicates of ESC Hi-C libraries used in this study. The plots reveal the expected relationship between interactions and genomic distance at increasing length scales. (f) Contact probability as a function of the genomic distance in logarithmic bins (without dividing by bin size). Lines represent the values from biological replicates of ESCs or the mean value from NSC Hi-C data. Enrichment is observed/total contacts. (g) Number of TADs identified in ESCs and NSC, separated by compartment. (h) Distance between NSC and ESC TAD borders compared to distance between two sets of randomly selected genomic intervals. (i) Comparison of scaled ESC and NSC insulation calculated at three region sizes (50, 300 and 500 kb) across the borders of ESC TADs. Profiles calculated in 1 kb bins in the 150 kb region flanking borders ('B') and smoothed. (j) Aggregate Hi-C maps in 50 kb region centred on ESC or NSC convergently bound CTCF sites (left) and distribution of the shaman scores in 20 kb region centred around the same convergent CTCF sites. (k) Histogram of detected TAD sizes for each dataset (l) Distribution of intra-TAD Shaman interaction scores as a function of genomic distance in all ESC and NSC TADs. (m) Aggregate Hi-C maps of ESCs grown in different conditions and thus representing different states of functional pluripotency. TAD borders from ESCs in 2i conditions (upper half) and FCS conditions (lower half) reveal increased inter-TAD interactions in 2i ESCs of ground state pluripotency (naïve). (n) Distribution of observed insulation at borders in ESC,2i (red) and ESC, FCS (blue). Central bar represents the median with boxes indicating the upper and lower quartiles. (o) Aggregate Hi-C maps of size-selected (30th-70th percentiles) TADs from both ESC populations showing increased intra-TAD interactions in ESC FCS cells. (p) Distribution of mean interaction score between 20 kb regions centered on intra-TAD border pairs in the two ESC populations. (q) Distribution of TAD connectivity defined as in the Methods for the two ESC populations. (r-u) Analysis of previously published high-resolution cellular differentiation model[1], revealing changes to (r, s) insulation and (t,u) TAD connectivity as a function of lineage commitment.

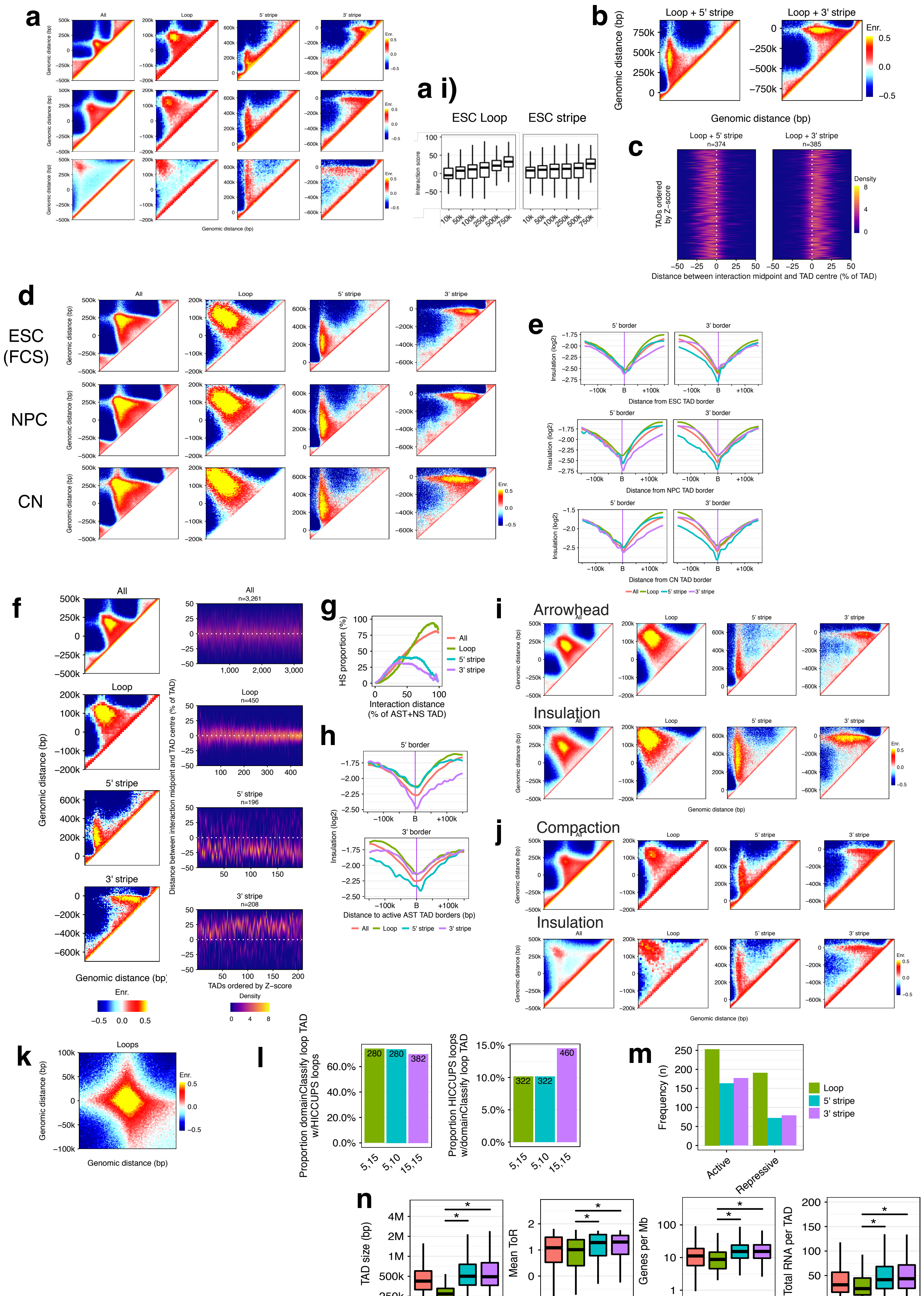

# SUPPLEMENTARY FIGURE 2

**Supplementary Figure 2.** (a) In support of Fig. 2b, aggregate Hi-C profiles for the shortest 30% (bottom) or largest 30% (top) of ESC classified TADs. All plots are shown as in Fig. 2b, with a resolution of 10kb. (a, i) Distribution of intra-TAD Shaman interaction scores as a function of genomic distance in classified ESC loop (left) or stripe (right) TADs. (b) Aggregate Hi-C contact maps of ESC TAD classes with enriched high-scoring contacts in both the loop and stripe TAD sectors. (c) Distance between high-scoring interactors relative to TAD center for the TADs in (b). Supporting data to validate the existence and properties of stripe TADs in multiple datasets and using different TAD segmentation methods. (d) High-resolution Hi-C data [1] was processed through domainClassifyR. Shown are the aggregate Hi-C plots for the classified and size-selected TADs in ESC, NPC and CN. The 'All' TAD set was size selected for the 40-60%ile and classified TADs were not size-selected. (e) Insulation profiles for the TAD classes identified from the Bonev Hi-C datasets (above), as in Fig 2e. Plotted is the mean contact insulation (300 kb band) within 150 kb of either the 5' or 3' TAD borders ('B' on the x-axis) and grouped according to TAD class. (f) Characterisation of classified AST TADs [2]. Shown are both the aggregate Hi-C contact maps for size-selected TADs and distance between high-score interactor relative to TAD centre. (g) Distribution of distances between high-scoring contact pairs in each class from AST as described in Fig 2d. (h) Contact insulation across both the 5' and 3' TAD borders of each class calculated using a 300 kb region size ASTs as described in Fig 2e. (i, j) Supporting data to show that use of different TAD segmentation methods does not preclude identification of TAD classes using domainClassifyR. Aggregate plots of classified HCT116 TADs where TADs were identified using (top) Arrowhead [3] or (bottom) Insulation [4]. (j) Aggregate plots of classified ESC TADs where TADs were defined using (top) Compaction [5] or (bottom) Insulation [4]. (k) Aggregate analysis of the 3,170 HCT116 loops identified by [6] showing a 200kb region around all loops at 2kb resolution. (l) (Right panel) Proportion of HCT116 classified TADs (using domainClassifyR) that intersect a HICCUPS-annotated loop. Proportions shown are of total classified TADS, with number of TADs in each class shown. (Left panel) Proportion of HICCUPS loops (n=3,170) that intersected an annotated TAD. Three Z-score threshold pairs are shown. (m) Number of classified ESC TADs identified in either genomic compartment using UCSC LaminB1 ESC track. (n) Epigenomic characteristics of classified active-compartment ESC TADs - TAD size, mean timing of replication, number of genes normalised to TAD size and total RNA-seq signal.

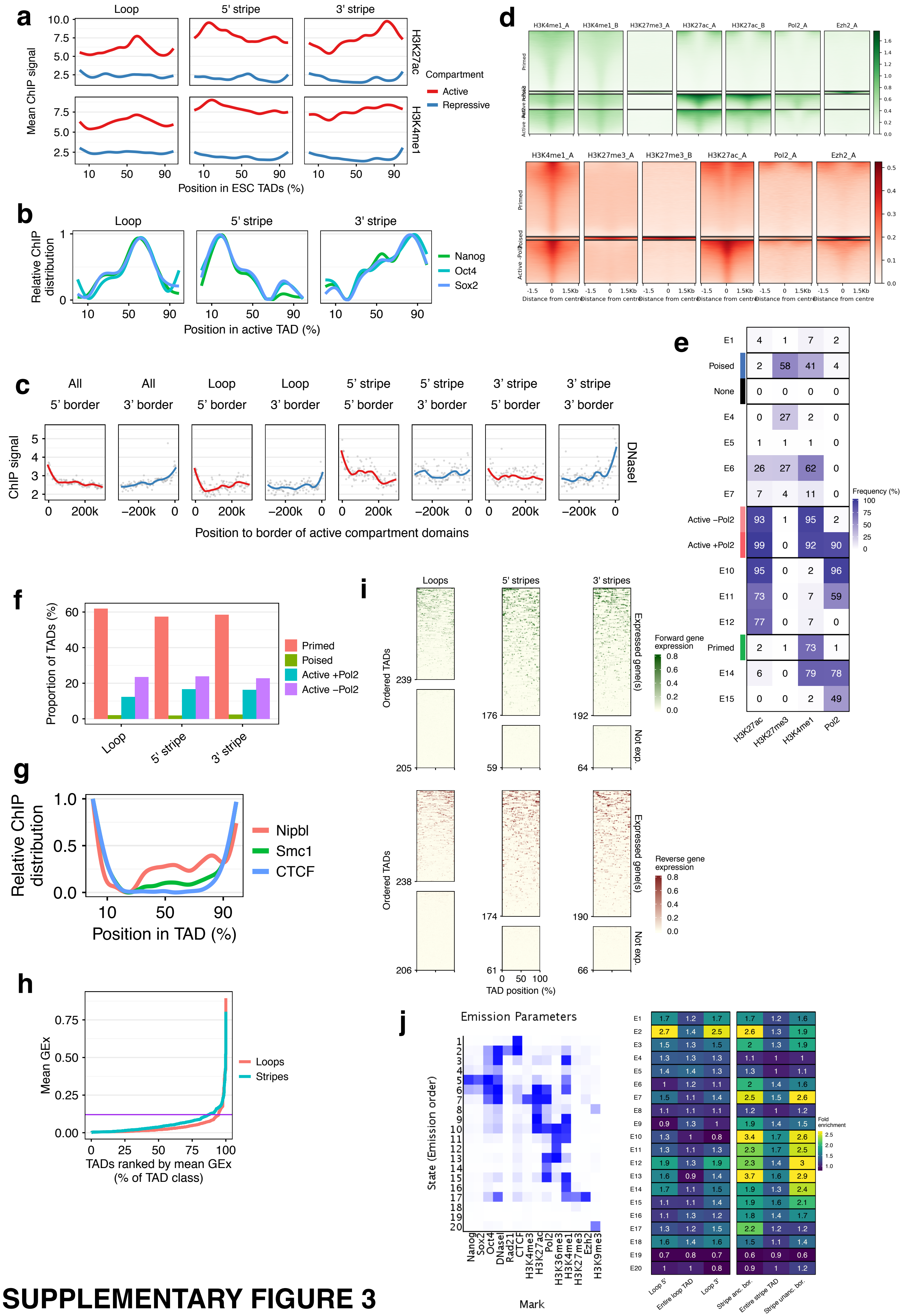


# SUPPLEMENTARY FIGURE 3

**Supplementary Figure 3.** (a) Scaled ChIP-seq signal distributions of enhancer histone marks (H3K4me1, H3K27ac) across ESC TAD classes, separated by compartment and shown as the mean signal. (b) Scaled relative ChIP-seq signal distributions of ESC transcription factors (Nanog, Oct4, Sox2) across ESC TAD classes. (c) ChIP-seq signal profile for DNase I in ESCs (2i) (ENCODE) across classified TAD borders, showing increased accessibility at anchored stripe borders. (d) deepTools visualisation of ESC (green) and NSC (red) genomic segments and the set of ChIP-seq tracks used to run ChromHMM. Signal is normalised using counts per million. (e) ChromHMM emission table highlighting the datasets used to name identified chromatin groups. (f) Proportion of ESC classified TADs containing one or more ChromHMM chromatin state. (g) Scaled relative profiles of ChIP-seq signal distributions of NIPBL, SMC1 and CTCF across all ESC TADs. (h) TADs were grouped as containing expressed genes if the mean RNA-seq signal value was above 0.12 (defined using h). (i) Mean RNA-seq signal in loop or stripe domains ranked by signal in TAD class. Genes were separated into those which were expressing and Forward oriented (Top row) or expressing and Reverse oriented (Bottom row) and their distribution was scaled across classified ESC TADs using deepTools. (j) (Right panel) An extended ChromHMM emission matrix showing 20 emission states using additional ChIP-seq data for CTCF, Rad21, Oct4, Nanog, Sox2 and histone modifications and DNaseI hypersensitivity in ESC. (Left panel) The enrichment of the extended states with respect to ESC TAD borders of loop (left panel) or stripe domains (right panel) was then calculated using ChromHMM Overlap Enrichment. Fold enrichment values are shown. Analysis as in Fig 3d. For information of the publcally available datasets used herein, refer to Supplementary Table S2.

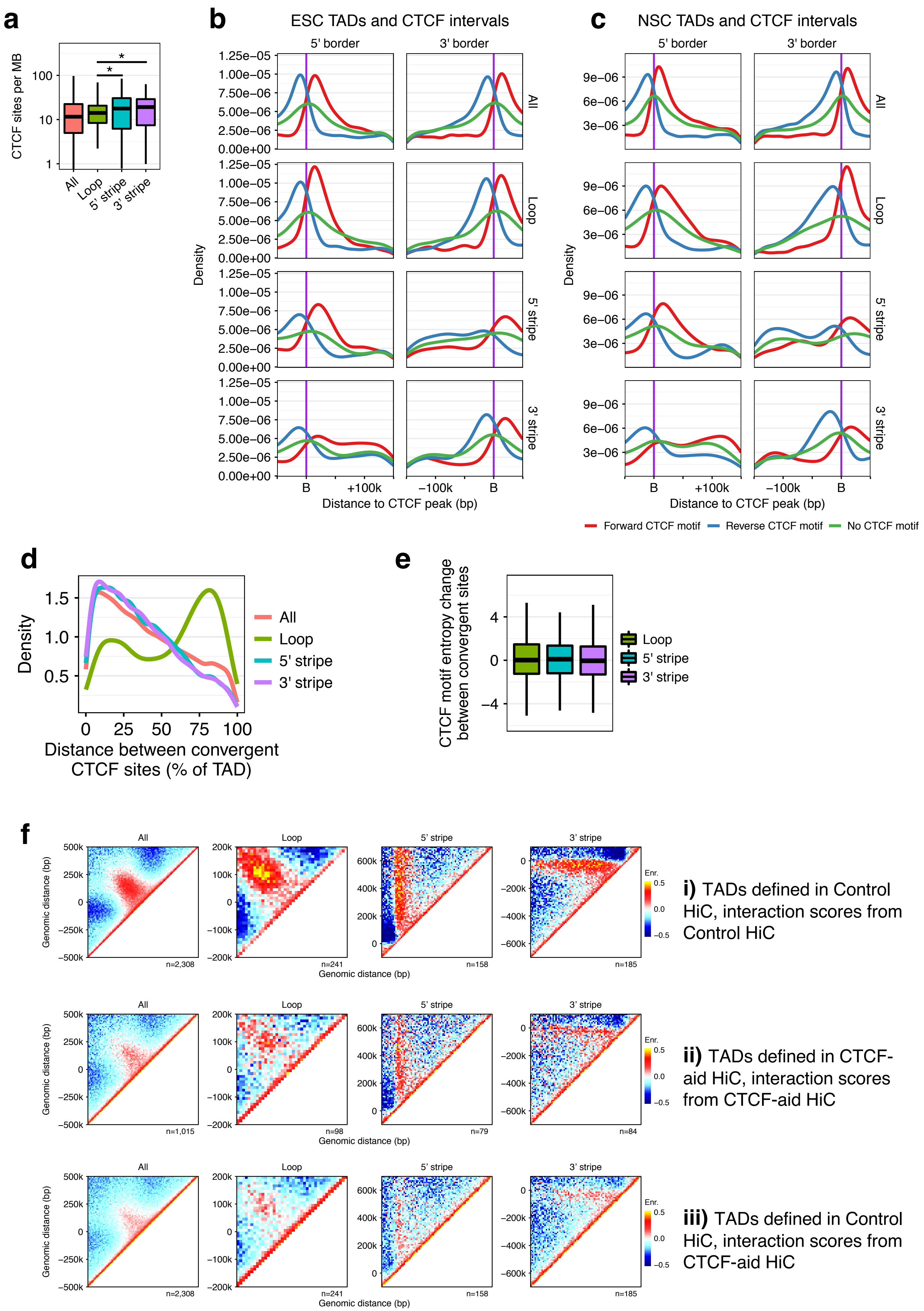


# SUPPLEMENTARY FIGURE 4

**Supplementary Figure 4.** (a) Number of CTCF sites in ESC classified TADs normalised by TAD size. (b) Density distributions of CTCF peak distance to either the 5’ or 3’ TAD border based on motif orientation and TAD class in ESCs. CTCF peaks were grouped according to the presence or absence of a forward (red) or reverse (blue) CTCF motif using FIMO in a genomic distance range of 50 kb upstream of the TAD border (‘B’ on the x-axis) to 150 kb within the TAD. Figure 4d from the main text is the same data but represented as an enrichment value. (c) Same as (b) but for NSCs. (d) Distribution of distance between convergent CTCF sites in ESC TAD classes. Figure 4e from the main text is the same data but represented as a scaled density for comparison purposes. (e) Delta CTCF motif entropy between convergent CTCF sites in ESC TAD classes. (f) Hi-C data from control or CTCF-AID-depleted ESCs [7] was downloaded and TADs classified using domainClassifyR. Panel i) shows the aggregate plots from classified TADs in the control ESC dataset revealing the same TAD classes as in other analysis herein. Panel ii) represents TAD classes from CTCF-depleted ESCs and the interactions scores from the same data. Please note that very few TADs from the mutant Hi-C contained enough data to be classified. The analysis shown here required a reduction in the minimum number of high-scoring interactions/TAD, removing the score threshold and plotting all profiles irrespective of TAD size into the aggregate plots. Panel iii) shows TADs defined in Control Hi-C and interactions scores from CTCF-depleted ESCs. Changes to TAD architecture are observable in both loop and stripe domains, suggesting an importance of CTCF in stripe architecture.

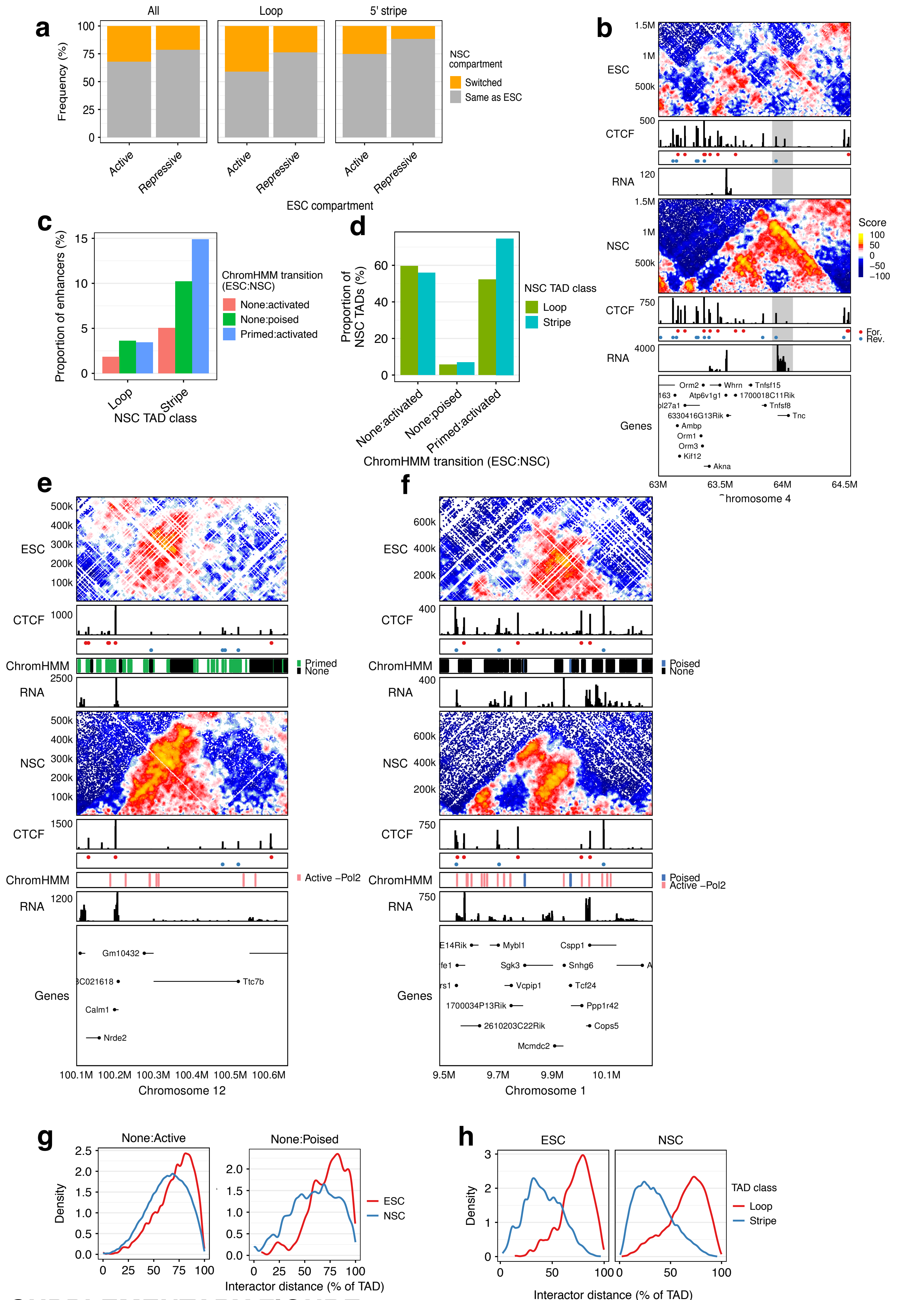


# SUPPLEMENTARY FIGURE 5

**Supplementary Figure 5.** (a) TAD classes were determined in ESC and NSCs and their compartment distribution analysed between the cell types. Orange represents ESC TADs which switch compartments in NSCs. (b) Genome topology changes during differentiation. *Tnc* has roles in neuronal development and growth and is not expressed in ESCs where it is contained within a poorly defined region. In NSCs, TAD structure is reinforced and anew prominent insulation site emerges. As with Amer2 (Figure 5d), the CTCF binding profile is very similar between the cell types, however the new insulation site occurs at the highly expressed *Tnc* gene. Layout and tracks as in Figure 5. (c) ChromHMM was used to define ESC- or NSC-specific enhancer element transitions and their proportion with respect to NSC TAD class determined. Results here are as in Figure 5e but include the ESC none:NSC poised enhancer transition (green bars). (d) Analysis as in (c) but as proportion of classified NSC TADs that contain a certain type of enhancer transition. (e) Lineage-specific enhancer activation contributes to the 'emergence' of stripe conformations. For example, (e) an ESC loop domain on chromosome 12 contains primed or none chromatin states throughout the TAD. In NSCs, the TAD structure is reinforced and associated with a stripe conformation. Activated enhancer elements (containing Pol2 marks) emerge towards the anchored border of the stripe domain. (f) In another example, a loop domain in ESCs is associated with mostly none and a few poised elements. New insulation and the appearance of stripe conformations emerge in NSCs alongside active chromatin states. (g) Contact distribution profile from chromatin segments associated with None-Active (left panel) or None-Poised (right panel) transitions in ESC (red) or NSC (blue), calculated exactly as in Figure 5f). (h) Contact distribution of loops (red) or stripes (blue) in ESCs (left panel) or NSCs (right panel). Data for NSCs is the same as the overlaid grey lines shown in Figure 5f for comparison purposes there.

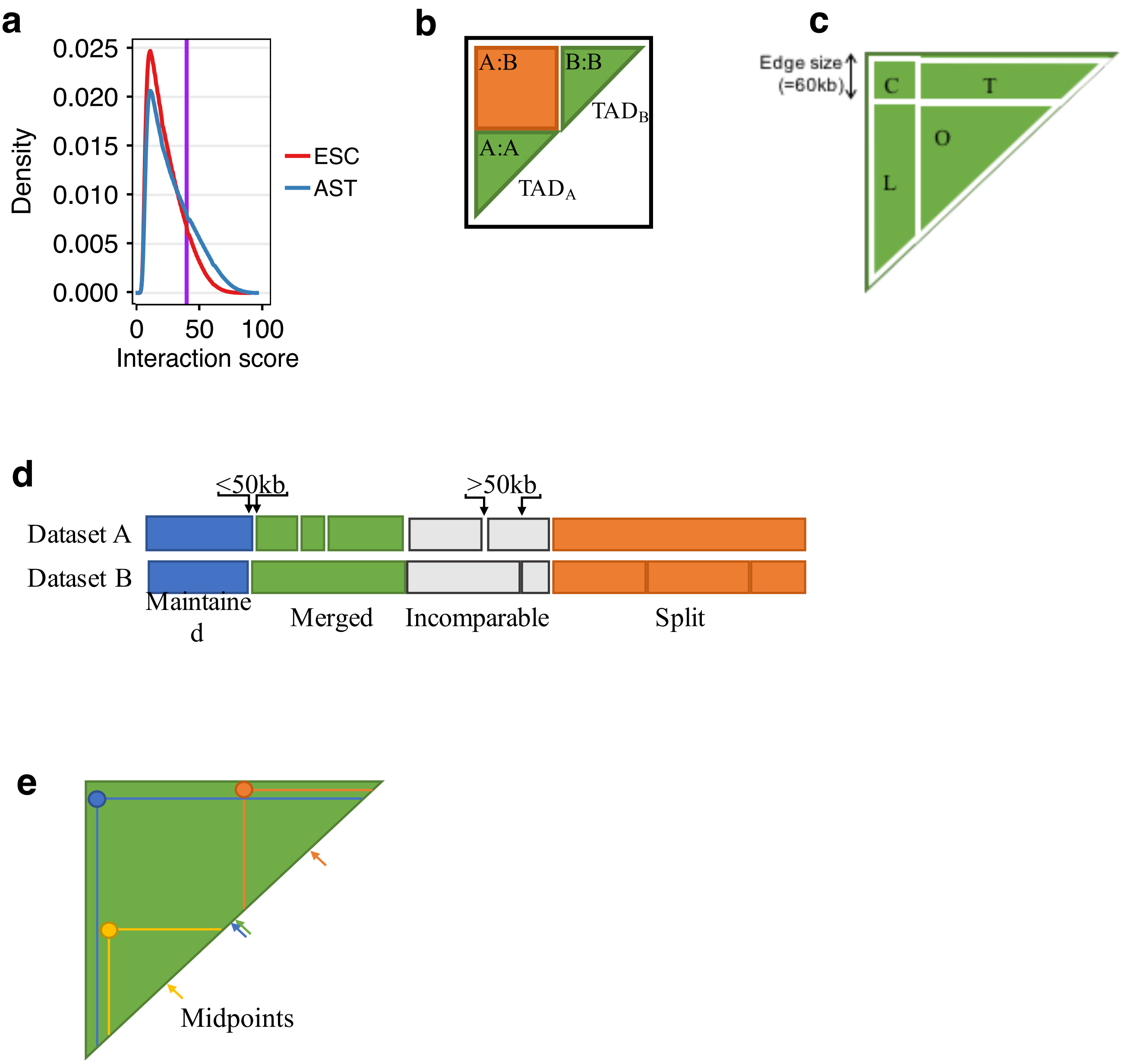


**Supplementary Figure 6. Related to Methods section. Classification and comparison of TAD structures.** (a) Distribution of contact scores within a TAD in ESC and AST. Scores >40 (purple vertical line) were used for TAD classification. Scores of 40 or greater represent the 93.5$^{th}$ percentile in our ESC and 90$^{th}$ percentile in our NSC dataset. (b) Defining TADs in 2D genome coordinates. (c) Schematic of sectors defined in TADs for downstream classification. (d) Schematic of TAD comparisons. (e) Schematic of a TAD (green) and interactions (coloured dots) showing the midpoints of the interactions corresponding coloured arrows) or the TAD (green arrow).

**Read statistics**

| Cell type | Sample ID | Library | Lane | Total Sequenced Reads | Uniquely mapped Reads | Total Contact Pairs |
|---|---|---|---|---|---|---|
| Embryonic Stem Cells (ESC), cultured in 2i conditions, this study. | **Hi-C_ESC_1** | **ESC_1_tech rep 1** | 1 | 810,335,240 | 512,885,166 | 145,581,280 |
| | | **ESC_1_tech rep 2** | 2 | 811,021,256 | 512,510,548 | 145,042,543 |
| | **Hi-C_ESC_2** | **ESC_2_tech rep 1** | 1 | 800,277,424 | 535,509,094 | 170,864,492 |
| | | **ESC_2_tech rep 2** | 2 | 799,123,000 | 536,256,887 | 170,030,220 |
| | **TOTAL** | | | **3,220,756,920** | **2,097,161,695** | **631,518,535** |
| NS cells (NSC) Sofueva et al. 2013 (GSM1192202) | **TOTAL** | | | **1,918,177,452** | **1,384,690,979** | **494,211,806** |
| Astrocytes (AST) Sofueva et al. 2013 (GSM1192209) | **TOTAL** | | | **1,249,958,151** | **937,468,613** | **374,987,445** |

**Supplementary Table 1.** Read Statistics for the Hi-C datasets prepared for and used in this study. Details of the Hi-C library processing steps and an analysis of Hi-C library quality control can be found in the Methods section. Shown are the read statistics for the Hi-C libraries in this study. Sequences and alignments are shown as individual reads while Contacts (used for Hi-C matrix generation) are represented as pairs.

| Data type | Author | Description for this study | Study | Accession(s) |
|---|---|---|---|---|
| Hi-C | Sofueva, 2013 | AST | GSE49017 | GSM1192209, 10 |
| | Sofueva, 2013 | NSC | GSE49017 | GSM1192202, 03 |
| | Bonev, 2017 | NPC | GSE96107 | GSM2533822 |
| ChIP-seq | Bonev, 2017 | NSC H3K4me1 | GSE96107 | GSM2533862, 63 |
| | | NSC H3K27ac | GSE96107 | GSM2533868, 69 |
| | | NSC H3K27me3 | GSE96107 | GSM2533870, 71 |
| | | NSC CTCF | GSE96107 | GSM2533858, 59 |
| | | NSC Input | GSE96107 | GSM2533874, 75 |
| | Huang, 2017 | NSC Pol2 | GSE89573 | GSM2384786 |
| | | NSC Input | GSE89573 | GSM2384787 |
| | Kundu, 2017 | NSC Ezh2 | GSE89929 | GSM2393590 |
| | | NSC H3K27me3 | GSE89929 | GSM2393591 |
| | | NSC Input | GSE89929 | GSM2393594, 95 |
| | Beagan, 2017 | ESC CTCF | GSE85185 | GSM2259905 |
| | | ESC Input | GSE85185 | GSM2259906 |
| | Buecker, 2014 | ESC H3K4me1 | GSE56098 | GSM1355160, 61 |
| | | ESC H3K27ac | GSE56098 | GSM1355158, 59 |
| | | ESC Input | GSE56098 | GSM1355163, 64 |
| | Dorighi, 2017 | ESC H3K4me1 | GSE98063 | GSM2586537, 38 |
| | | ESC H3K27ac | GSE98063 | GSM2586543, 44 |
| | | ESC Pol2 | GSE98063 | GSM2586549, 50 |
| | | ESC Input | GSE98063 | GSM2586555 |
| | Marks, 2012 | ESC Ezh2 | GSE23943 | GSM590132 |
| | | ESC H3K4me3 | GSE23943 | GSM590112 |
| | | ESC H3K9me3 | GSE23943 | GSM850407 |
| | | ESC H3K27me3 | GSE23943 | GSM590120 |
| | | ESC H3K36me3 | GSE23943 | GSM590120 |
| | | ESC Pol2 | GSE23943 | GSM758173 |
| | Kagey, 2010 | ESC Nipbl | GSE22562 | GSM560349, 50 |
| | | ESC Smc1 | GSE22562 | GSM560341, 42 |
| | Whyte, 2013 | ESC Nanog | GSE44286 | GSM1082342 |
| | | ESC Oct4 | GSE44286 | GSM1082340 |
| | | ESC Sox2 | GSE44286 | GSM1082341 |
| | UCSC | ESC LaminB1 | | |
| | | NSC LaminB1 | | |
| RNA-seq | Sofueva, 2013 | NSC Gene Expression | GSE48965 | GSM1187679 |
| | Marks, 2012 | ESC Gene Expression | GSE23943 | GSM850403 |
| Repli-seq | ENCODE | ESC timing of replication | GSE95091 | |
| Dnase-seq | ENCODE | DNaseI | GSE37074 | GSM1014154 |

**Supplementary Table 2.** Publically available datasets used in this study.

## Supplementary References.


1. Bonev, B. *et al.* Multiscale 3D Genome Rewiring during Mouse Neural Development. *Cell* **171,** 557.e1–557.e24 (2017).
2. Sofueva, S. *et al.* Cohesin-mediated interactions organize chromosomal domain architecture. *EMBO J.* **32,** 3119–3129 (2013).
3. Rao, S. S. P. *et al.* A 3D map of the human genome at kilobase resolution reveals principles of chromatin looping. *Cell* **159,** 1665–1680 (2014).
4. Nagano, T. *et al.* Cell-cycle dynamics of chromosomal organization at single-cell resolution. *Nature* **547,** 61–67 (2017).
5. Vietri Rudan, M. *et al.* Comparative Hi-C reveals that CTCF underlies evolution of chromosomal domain architecture. *CellReports* **10,** 1297–1309 (2015).
6. Rao, S. S. P. *et al.* Cohesin Loss Eliminates All Loop Domains. *Cell* **171,** 305–309.e24 (2017).
7. Nora, E. P. *et al.* Targeted Degradation of CTCF Decouples Local Insulation of Chromosome Domains from Genomic Compartmentalization. *Cell* **169,** 930–933.e22 (2017).
